# A bacterial route for folic acid supplementation

**DOI:** 10.1186/s12915-018-0534-3

**Published:** 2018-06-15

**Authors:** Claire Maynard, Ian Cummins, Jacalyn Green, David Weinkove

**Affiliations:** 1 Department of Biosciences, Durham University, Stockton Road, Durham, DH1 3LE UK; 2 Midwestern University, Illinois, Downers Grove, IL 60515 USA; 3 Biophysical Sciences Institute, Durham University, South Road, Durham, DH1 3LE UK

## Abstract

**Background:**

To prevent folate deficiencies, many countries supplement various foodstuffs with folic acid. This compound is a synthetic oxidised folate that differs from naturally occurring reduced folates in its metabolism and uptake. Notably, safety reviews of folic acid supplementation have not considered interactions with gut bacteria. Here, we use the *Caenorhabditis elegans – Escherichia coli* animal– microbe model to examine a possible bacterial route for folic acid uptake. It has been assumed that supplements are taken up directly by the worm, especially because *E. coli* is unable to take up folates. However, *E. coli*, like many other bacteria, can transport the folate breakdown product, para-aminobenzoate-glutamate (PABA-glu), via AbgT and use it for bacterial folate synthesis. This pathway may impact host health because inhibition of bacterial folate synthesis increases *C. elegans* lifespan.

**Results:**

Folic acid supplementation was found to rescue a *C. elegans* developmental folate-deficient mutant; however, a much higher concentration was required compared to folinic acid, a reduced folate. Unlike folinic acid, the effectiveness of folic acid supplementation was dependent on the *E. coli* gene, *abgT*, suggesting a bacterial route with PABA-glu uptake by *E. coli* as a first step. Surprisingly, we found up to 4% PABA-glu in folic acid preparations, including in a commercial supplement. Via breakdown to PABA-glu, folic acid increases *E. coli* folate synthesis. This pathway restores folate synthesis in a bacterial mutant defective in PABA synthesis, reversing the ability of this mutant to increase *C. elegans* lifespan.

**Conclusions:**

Folic acid supplementation in *C. elegans* occurs chiefly indirectly via bacterial uptake of breakdown products via *E. coli* AbgT, and can impact *C. elegans* development and longevity. Examining how folic acid supplementation affects bacterial folate synthesis in the human gut may help us to better understand the safety of folic acid supplementation.

**Electronic supplementary material:**

The online version of this article (10.1186/s12915-018-0534-3) contains supplementary material, which is available to authorized users.

## Background

The folate cycle involves a series of essential biosynthetic reactions known as one-carbon metabolism [1]. Folates are a family of molecules composed of three common elements: a central aromatic core derived from para-amino benzoic acid (PABA), a pterin ring that can be modified and a chain of one or more glutamates [2]. At each step of the folate cycle, an enzyme mediates a modification of the pterin ring of the bound folate, allowing the transfer of a chemical group containing one carbon atom (methyl, formyl, etc.) to or from the compound being synthesised [1]. This cofactor role results in folate molecules being recycled and thus they are only required in very small amounts.

Animals cannot synthesise folates and must acquire them from their diet or gut microbes. When these sources are insufficient, folate deficiency can lead to neural tube defects during human embryonic development [1]. The rate of these defects can be lowered by preconception supplementation with folic acid, an oxidised form of folate not found in nature. Mandatory fortification of flour with folic acid has successfully decreased the incidence of birth defects in many countries, including the US and Canada [3]. However, there are concerns that folic acid supplementation may have adverse effects on health, especially in older people [4–7], and there are many unknowns about the efficacy of uptake and biological utilisation of folic acid [8]. Despite these uncertainties, recent reviews of the evidence by experts acting for government public health bodies have concluded that the risks are minimal and have recommended the fortification of flour or other food products as a beneficial intervention [3, 9, 10].

None of the above safety reviews mention potential interactions with gut bacteria. Bacteria also require folates for biosynthesis. Many make their own folates, but folates can also be taken up from the environment. Little is known about how bacterial folate biochemistry affects the host. Inhibiting *Escherichia coli* folate biosynthesis, either by treatment with the drug sulfamethoxazole (SMX) or mutation of the PABA synthesis pathway (e.g., a *pabA* or *pabB* mutant), extends the lifespan of the nematode *Caenorhabditis elegans* that feeds on it [11–13]. While these interventions decrease the folate levels in both *E. coli* and *C. elegans*, there remains sufficient folate available to support normal growth of both organisms [12]. We have suggested that bacterial folate, at higher levels than need for growth, enables a bacterial activity that shortens the life of *C. elegans* and is independent of the folate status of *C. elegans* [13]. Thus, the folate status of gut bacteria may be important for host health.

To understand the interaction between bacterial and animal folates, we have developed a *C. elegans* folate deficiency model, a mutant in *gcp-2.1* and homologue of human GCPII, which is required for uptake of dietary polyglutamated folates [13]. The only way we have found of decreasing *C. elegans* folate enough to slow growth is by raising the *C. elegans gcp-2.1* mutant on SMX-treated *E. coli*. Under these conditions, worms show delayed development and infertility [13]. This phenotype can be prevented with 1–10 μM folinic acid, a reduced folate which can be absorbed directly by *C. elegans*, demonstrating that this defect is due to folate deficiency. In contrast, prevention with folic acid requires much higher concentrations (100 μM) [13]. We also discovered that, at high concentrations, folic acid can partially reverse the lifespan increase caused by inhibiting *E. coli* folate synthesis [12]. A possible explanation for this result might be the restoration of *E. coli* folate levels, but that would be a surprising conclusion because *E. coli* does not possess a folate transporter [14, 15]. However, it is known that *E. coli* can take up the folic acid breakdown product PABA-glu through the transporter AbgT and catabolise it to PABA [16]. PABA can also diffuse across *E. coli* membranes. Increases in bacterial PABA levels from either source can be used to synthesise folate. In this study, we examine whether these routes might explain the lifespan effects of folic acid.

**Fig. 1:**
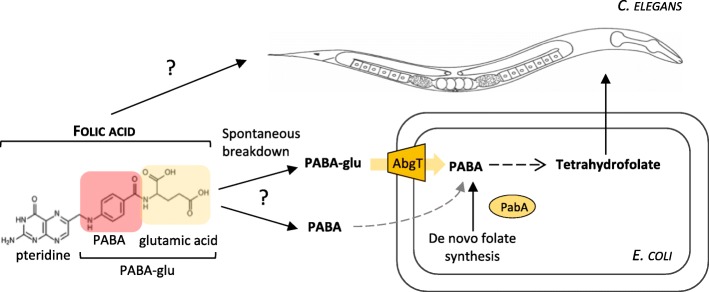
Schematic representing possible *C. elegans* and bacterial-dependent routes of folic acid supplementation. *C. elegans* depends on *E. coli* for folate. *E. coli* synthesizes reduced tetrahydrofolates *de novo* via the PABA pathway. The synthetic folate supplement, folic acid, is oxidised and must be enzymatically reduced into a tetrahydrofolate before it is bioavailable. *E. coli* cannot take up intact folic acid, but it can take up the breakdown product, PABA-glu. The inner membrane transporter AbgT transports PABA-glu, where it is cleaved intracellularly by a heterodimeric complex to generate PABA. Free PABA is then used to generate tetrahydrofolates. It is not clear whether this bacterial pathway is significant in the supplementation of an animal by folic acid

Here, we use a system in which the external media, microbe and animal can be carefully controlled, to ask whether folic acid is taken up directly by *C. elegans* or uptake of folic acid breakdown products by *E. coli* represents an important alternative route (Fig. 1). We show that the prevention of developmental defects of the *C. elegans* folate deficiency model by adding folic acid depends on the *E. coli* AbgT transporter, demonstrating that bacterial uptake is the major route of supplementation. Consistent with this route, we show folic acid increases *E. coli* folate levels through a pathway that involves uptake of PABA-glu by the AbgT transporter and that PABA-glu is present in folic acid preparations. We also find that this pathway can reverse the lifespan increase caused by inhibiting *E. coli* folate synthesis. In summary, we have uncovered an unappreciated breakdown product in folic acid supplements and a bacterial route of uptake that can increase host folate levels indirectly but might also increase bacterial toxicity, accelerating ageing.

## Results

### *E. coli* is required for folic acid supplementation to prevent a *C. elegans* folate deficiency

**Fig. 2:**
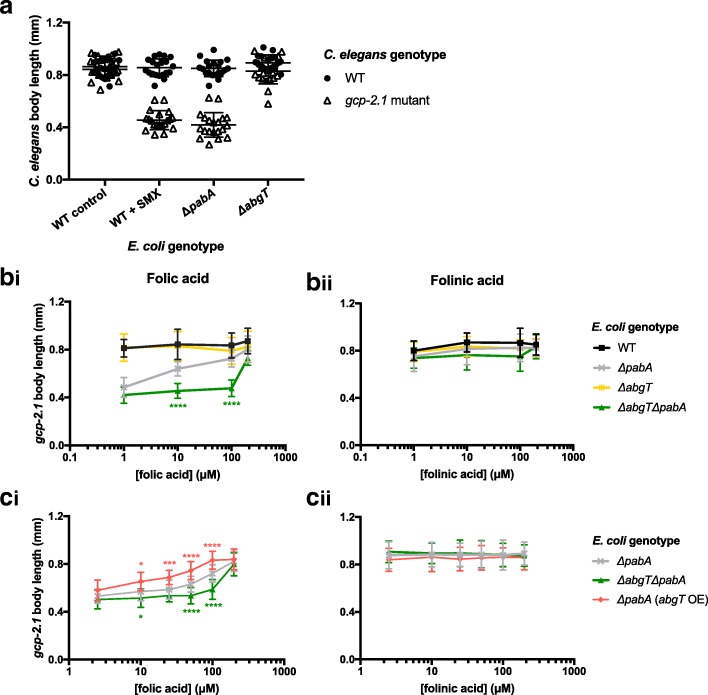
Folic acid supplementation prevents developmental growth defect of a *C. elegans* folate deficiency model via an *E. coli abgT*-dependent route. Body length of WT and *gcp-2.1* mutant *C. elegans* at L4 developmental stage raised on DM agar plates seeded with (**a**) WT *E. coli* (control), WT *E. coli* treated with 128 μg/mL SMX, *ΔpabA* mutant, and *ΔabgT* mutant, (**b**) *ΔpabA* mutant, *ΔabgT ΔpabA* double mutant, *ΔpabA* (*abgT* OE), and supplemented with increasing concentrations of folic and (**c**) folinic acid. Error bars represent standard deviation of *C. elegans* body length, where *n* ≥ 40. Asterisks (*) indicate test statistic of unpaired non-parametric Mann–Whitney *t* tests, where ****p < 0.0001, ***p < 0.001, **p < 0.01, *p < 0.05. By two-way ANOVA analyses, we find that there is a significant interaction effect of strain type (F = 102.67, *p* < 0.0001) and folic acid concentration (F = 123.55, *p* < 0.0001) on *C. elegans gcp-2.1* body length. Over-expression of *abgT* is conferred by transformation with a high copy number plasmid, pJ128. *ΔpabA* and *ΔabgT ΔpabA* strains are transformed with the empty vector, pUC19

In order to examine if *E. coli* is required for folic acid to prevent developmental defects in our *C. elegans* folate deficiency model, we tested whether the *E. coli abgT* genotype influenced the outcome of supplementation. Our folate deficiency model [13] is based on the measurable growth defect of the *C. elegans gcp-2.1(ok1004)* mutant during larval development, when it is grown on OP50 *E. coli* treated with 128 μg/mL SMX (Fig. 2a). In this study, we found a similar growth phenotype (Fig. 2a) when we grew this *C. elegans* mutant on an *E. coli* Δ*pabA* mutant (which cannot make its own folate because it cannot make the folate precursor PABA). To eliminate exogenous PABA, we grew the bacteria on a defined media (DM) containing only amino acids, salts, cholesterol and trace metals [13]. Mutation of *E. coli abgT* alone did not influence the growth of the *C. elegans gcp-2.1* mutant (Fig. 2a), because *de novo* folate synthesis is unaffected by the Δ*abgT* mutant. To examine the role of AbgT in the absence of endogenous folate synthesis, an *E. coli* Δ*abgT* Δ*pabA* double mutant strain was constructed. Developmental growth of *C. elegans gcp-2.1* mutants on the Δ*abgT* Δ*pabA E. coli* was delayed just as it was on the Δ*pabA* mutant. In response to folic acid supplementation over a 100-fold concentration range, a dose-dependent increase in body length was observed for *gcp-2.1* mutants on the *E. coli* Δ*pabA* mutant, which was not observed on the Δ*abgT* Δ*pabA* mutant (Fig. 2bi, asterisks indicate significant difference between Δ*pabA* and Δ*abgT* Δ*pabA*). In contrast to the 200 μM folic acid required to completely rescue growth of the *gcp-2.1* mutant worms on *E. coli* Δ*pabA* and Δ*abgT* Δ*pabA*, only 1 μM folinic acid was required, and supplementation was independent of *E. coli* genotype (Fig. 2bii), consistent with a direct route to the worm for folinic acid.

Overexpression of *abgT* in the *E. coli* Δ*pabA* mutant (Δ*pabA* (*abgT* OE)) increased *gcp-2.1* mutant body length at lower concentrations of folic acid, with a complete rescue achieved at 100 μM folic acid (Fig. 2ci). Consistent with the previous experiment, folinic acid rescued *gcp-2.1* mutant growth at a 100-fold lower concentration and independently of *abgT* expression (Fig. 2cii). Analysing the experiment by two-way ANOVA, we find that there is a significant interaction effect of *abgT* genotype (F = 102.67, *p* < 0.0001) and folic acid concentration (F = 123.55, *p* < 0.0001) on *C. elegans gcp-2.1* body length (Fig. 2ci). These results are consistent with folinic acid being taken up directly by the worm [17], and the major route of folic acid uptake requiring *E. coli* and the *E. coli* AbgT transporter.

### Folic acid supports growth *of E. coli pabA* mutants via *abgT*-dependent uptake of PABA-glu

**Fig. 3:**
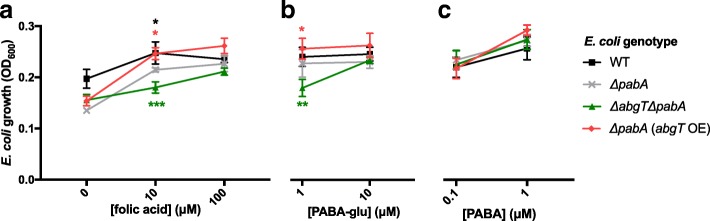
Folic acid supplement breakdown supports *E. coli* growth. Bacterial lawn growth, under PABA-depleted conditions, of *E. coli* WT, *ΔpabA*, *ΔabgT ΔpabA* and *ΔpabA (abgT* OE) on DM plates supplemented with (**a**) folic acid, (**b**) PABA-glu and (**c**) PABA as measured by OD_600_ after 4 days growth at 25 °C (see Methods). Each data point is the average of 8 plates. Error bars represent standard deviation. Asterisks denote the test statistic from Student’s *t* test comparison of means, where ***p < 0.001, **p < 0.01,*p < 0.05 compared to *ΔpabA* growth on the comparable condition

As AbgT is known to transport the folate breakdown product PABA-glu, the results above suggest that PABA-glu is available to *E. coli* following folic acid supplementation. To test this assertion, we conducted growth experiments using the *E. coli* Δ*pabA* mutant under conditions in which there was insufficient exogenous PABA available to support growth, namely growth on DM agar plates when seeded after several generations of growth in liquid DM. We assessed how the growth of the Δ*pabA*, Δ*abgT* Δ*pabA* and Δ*pabA* (*abgT OE*) strains could be restored with folic acid, PABA-glu or PABA when added to the media. We found that the growth restored by folic acid or PABA-glu depended on *abgT* expression; 10 μM folic acid rescued the growth of the Δ*pabA* mutant, whereas 100 μM was needed to achieve an equivalent rescue in the Δ*abgT* Δ*pabA* double mutant (Fig. 3). In the presence of 10 μM folic acid, growth of the Δ*pabA* strain over-expressing AbgT was greater than that of the Δ*pabA* mutant alone. Supplementation by PABA-glu had a similar effect to folic acid but at a 10-fold lower concentration (Fig. 3), consistent with results of experiments in liquid at 37 °C [16]. PABA, which can diffuse across biological membranes [18], rescued bacterial growth at nanomolar concentrations independently of *abgT* expression (Fig. 3). Overall, restoration of bacterial growth by folic acid can be largely explained by PABA-glu uptake by AbgT, while low concentrations of PABA present in folic acid preparations may explain the ability of high concentrations of folic acid to rescue the growth of the *E. coli* Δ*abgT* Δ*pabA* double mutant.

### Folic acid increases *E. coli* folate levels in an AbgT-dependent mechanism

**Fig. 4:**
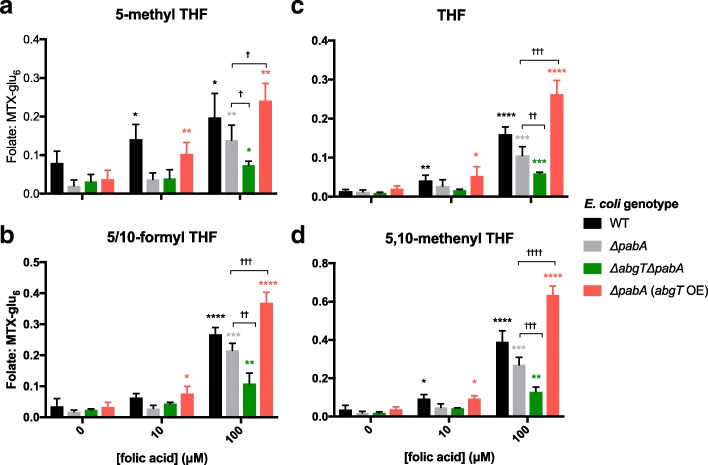
Folic acid increases *E. coli* folate levels via uptake of PABA-glu by AbgT. Levels of (**a**) 5-methyl THF-glu_3_, (**b**) 5/10formyl THF-glu_3_, (**c**) THF-glu_3_ and (**d**) 5,10 methenyl THF-glu_3_ in extracts of *E. coli* WT, *ΔpabA*, *ΔabgT ΔpabA* and *ΔpabA* (*abgT* OE) mutants supplemented with 10 μM and 100 μM folic acid. Extracts were made after 4 days of bacterial growth at 25 °C on solid agar plates. Folate counts from the LC-MS/MS were normalised by dividing by counts of an internal MTX-glu_6_ spike. Error bars represent standard error of the mean of at least four replicate samples per data point (Data in Additional file 3: Table S2). Asterisks indicate test statistic of unpaired parametric *t* tests with Welch’s correction, where ****p < 0.0001, ***p < 0.001, **p < 0.01,*p < 0.05. Pairwise comparisons between *ΔpabA* and *ΔabgT ΔpabA*, *ΔpabA* (*abgT* OE) at 100 μM folic acid are indicated by †

In order to verify that *E. coli* growth following folic acid supplementation is attributable to restored bacterial folate synthesis, we used LC-MS/MS (see Methods) to detect levels of individual *E. coli* tetrahydrofolates (THFs) under the conditions used in the above experiment. Levels of the most detectable, and thus likely most abundant, THF species (5-methyl THF-glu_3_, 5/10-formyl THF-glu_3_, THF-glu_3_ and 5,10-methenyl THF-glu_3_) are presented in Fig. 4. With supplementation of 10 μM folic acid, folate levels in Δ*pabA* and Δ*abgT* Δ*pabA* were not significantly increased compared to their non-supplemented controls. In contrast, in wild-type (WT) and Δ*pabA* (*abgT* OE) strains, addition of 10 μM folic acid resulted in significantly higher levels of folates. In response to 100 μM folic acid, folate levels increased in all strains compared to their non-supplemented controls, where the scale of increase was dependent on *abgT* expression (Fig. 4). At 100 μM folic acid, folate levels were highest in Δ*pabA* (*abgT* OE) followed by WT, Δ*pabA*, and finally lowest in the Δ*abgT* Δ*pabA* double mutant (Fig. 4). The *abgT* genotype had a strong effect on the folate levels of the Δ*pabA* strains at 100 μM folic acid (Fig. 4, pair-wise comparisons between Δ*pabA* and the Δ*abgT* Δ*pabA* and Δ*pabA* (*abgT* OE) strains are indicated by crosses). In summary, folic acid supplementation was found to increase *E. coli* folate levels in an *abgT-*dependent mechanism.

### Folic acid preparations contain PABA-glu and PABA

**Fig. 5:**
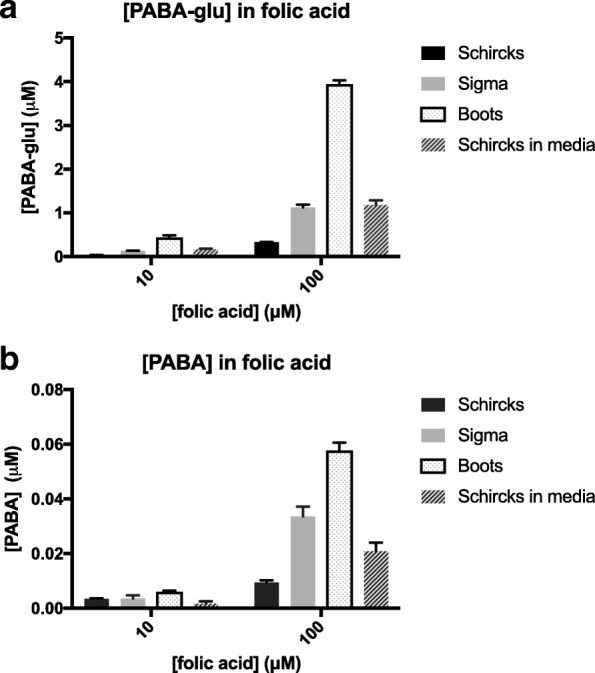
Folate preparations contain PABA-glu and PABA. Concentrations of (**a**) PABA-glu and (**b**) PABA as determined by LC-MS/MS in 10 and 100 μM folic acid preparations. Samples were folic acid from Schircks, Sigma, Boots and Schircks folic acid after addition to the agar media and incubation for 4 days at 25 °C. Error bars represent standard deviation over triplicate independent preparations. Full data in Additional file 4: Table S3

Together, the data presented here indicate that the main route of *C. elegans* folic acid supplementation is indirect via *E. coli* uptake of PABA-glu and PABA. We used LC-MS/MS to test for the presence of these breakdown products in folic acid preparations from Schircks (used in all other experiments in this study), Sigma Aldrich, and Boots, a UK retailer of supplements. We also tested for further folic acid breakdown under the experimental conditions used here by analysing extracts from agar media supplemented with Schircks folic acid and incubated at 25 °C for 4 days. We detected PABA-glu in all three folic acid sources at between 0.3% (Schircks) and 4% (Boots, Fig. 5). Under the conditions used for *C. elegans* experiments, PABA-glu increased to 1.18%, suggesting further breakdown. PABA was found at between 0.01% (Schircks) and 0.06% (Boots, Fig. 5); this level of PABA in folic acid preparations may explain why folic acid can increase folates in a Δ*abgT* Δ*pabA* double mutant.

### Folic acid shortens *C. elegans* lifespan via AbgT-dependent uptake of PABA-glu

**Fig. 6:**
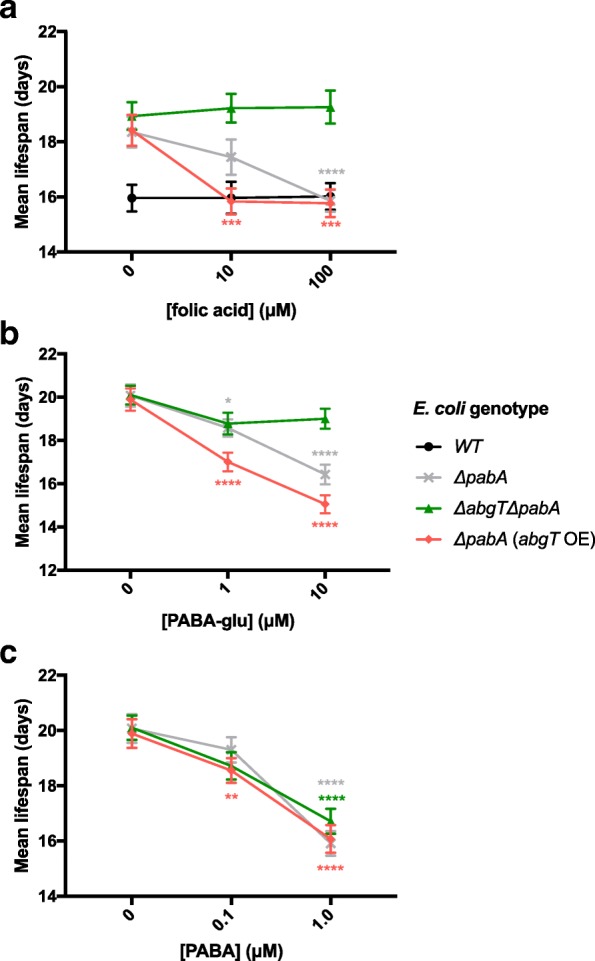
Folic acid shortens *C. elegans* lifespan via an *E. coli abgT*-dependent route during adulthood. Mean lifespan of *glp-4(bn2*) *C. elegans* maintained from day 1 of adulthood on WT (**a** only), *ΔpabA*, *ΔabgT ΔpabA* or *ΔpabA* (*abgT* OE) mutant *E. coli* with supplementation of (**a**) folic acid, (**b**) PABA-glu and (**c**) PABA. Error bars represent standard error. Asterisks denote the Log-rank non-parametric statistical test of survival, where ****p < 0.0001, ***p < 0.001, **p < 0.01, *p < 0.05, compared to lifespan on the non-supplemented condition of the same strain. Full lifespan data in Additional file 1: Table S1

Inhibiting bacterial folate synthesis, without affecting bacterial growth, is known to increase *C. elegans* lifespan [12, 13]. It was therefore hypothesized that, if folic acid increased bacterial folate synthesis, it may shorten *C. elegans* lifespan under conditions where bacterial folate synthesis was inhibited. Consistent with our previous findings [13], we find that *C. elegans* maintained on any *E. coli* Δ*pabA* mutant are long-lived compared to *C. elegans* fed WT *E. coli* (Fig. 6, Additional file 1: Table S1), whereas the Δ*abgT* mutation alone had no impact on *C. elegans* lifespan (*p* = 0.4312, Additional file 2: Figure S1b). Further, 10 μM folic acid was found to decrease *C. elegans* lifespan on Δ*pabA E. coli* by 9.4% (*p* = 0.0052), and to decrease it even further on Δ*pabA E. coli* over-expressing *abgT* (by 16.3%, *p* < 0.0001, Fig. 6a), whereas it had no effect on lifespan on the *ΔabgT ΔpabA* double mutant (*p* = 0.1901, Fig. 6a). In contrast, 100 μM folic acid decreased the lifespan on Δ*pabA E. coli* by 23.9% (*p* < 0.0001), whereas this concentration only shortened the lifespan on the Δ*abgT* Δ*pabA* double mutant by 4.7% (*p* = 0.0467, Fig. 6a). Lifespans on media supplemented with PABA-glu showed an *abgT*-dependent response similar to that observed with folic acid supplementation, but at a 10-fold lower concentration (Fig. 6b). In contrast, PABA supplementation shortened *C. elegans* lifespan in all cases independently of *abgT* expression (Fig. 6c), consistent with the ability of PABA to rescue *E. coli* folate production in a Δ*abgT* Δ*pabA* double mutant. Together, these results suggest that folic acid shortens *C. elegans* lifespan on folate-depleted *E. coli* via AbgT-dependent uptake of PABA-glu.

## Discussion

In this study we have found that a major route of uptake for the synthetic supplement folic acid by *C. elegans* is via *E. coli* rather than directly via the worm. Why is folic acid not taken up directly by *C. elegans* with the same affinity as folinic acid? One possible explanation is that FOLT-1, the only characterised *C. elegans* folate transporter, is a reduced folate carrier with specificity for folinic acid [17]. In humans, folic acid is also taken up in a different manner to natural reduced folates, with the major route thought to be via the protein-coupled folate transporter in the small intestine [19]. A *C. elegans* PCFT homologue has been identified, but its affinity for folic acid has not been characterized [20]. In addition, a homologue of the human folate receptor has been characterised in *C. elegans* [21]. This study does not rule out that direct routes for folic acid exist in *C. elegans*, but indicates that they are inferior to the *E. coli* route.

**Fig. 7:**
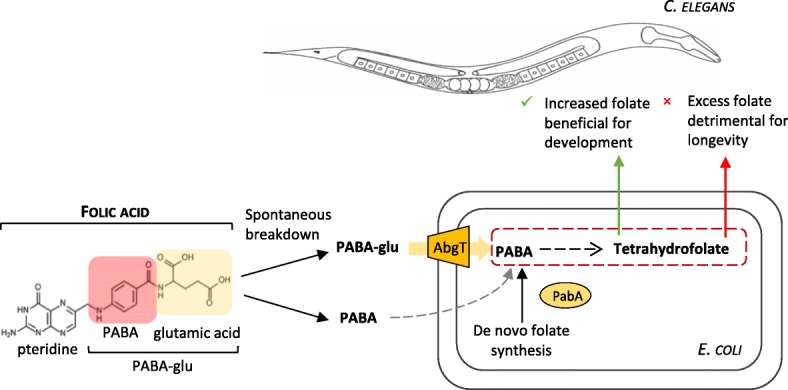
Schematic of the impact of folic acid supplementation on *C. elegans* via indirect uptake of breakdown products by *E. coli.* Folic acid is not taken up well by *C. elegans* directly. We find that the major uptake of folic acid by *C. elegans* is dependent on its breakdown into PABA-glu and uptake by the *E. coli* AbgT transporter. This route increases bacterial folate synthesis in both WT and *ΔpabA* mutant *E. coli.* Under conditions of folate deficiency (*ΔpabA* mutant *E. coli*), increasing bacterial folate via this route is beneficial for *C. elegans* development. During *C. elegans* adulthood, this route has a negative impact on longevity as it promotes a bacterial folate-dependent toxicity

We have found that *E. coli* uptake of folic acid is dependent on its spontaneous breakdown into PABA-glu, which can be taken up by the *E. coli* PABA-glu transporter, AbgT. This transporter, which as far as we know is the only *E. coli* transporter for PABA-glu, has evolved to salvage PABA-glu from the breakdown of natural folates, and is found in many bacteria in the human gut microbiota [22]. To our knowledge, this is the first study to show that this bacterial gene, and its level of expression, can influence the biology of the host, both by providing a route for folic acid to prevent host folate deficiency (Fig. 2), but also because it can lead folic acid to shorten the *C. elegans* lifespan when worms are cultured on folate-depleted *E. coli* (Fig. 6). The increase in folate synthesis caused by folic acid supplementation leads to a bacterial activity/toxicity that is harmful to the worm over the long term [13] (Fig. 7).

It is possible that this bacterial route for folic acid exists in humans. As far as we are aware, no other studies have tested folic acid supplements for the presence of PABA-glu or PABA, but several studies have reported issues with the stability and dissolution of commercial folic acid supplements [23, 24]. In light of these issues, manufacturers have adopted a policy of ‘overages’ in order to ensure sufficient folic acid is released in the small intestine following ingestion [25]. The presence of PABA-glu and PABA in a commercially available folic acid source (Fig. 6), combined with the instability of folic acid at the low pH conditions of the stomach [26–28], makes it likely that PABA-glu and PABA will be available to the gut microbiota following supplementation. PABA has been identified as a human faecal excretory product after ingestion of folic acid [29]. Furthermore, studies in rodents [30] and piglets [31] have demonstrated that infusion of labelled PABA into the cecum results in the incorporation of bacterially synthesized folate in host tissues*.* Thus, the literature suggests that the components for such a route exist in humans, but the importance of this route is yet to be determined. We detected this route in *C. elegans* because of the poor bioavailability of folic acid in our folate deficiency model. Folic acid is taken up well by humans and leads to increases in serum folate levels, but circulating folic acid is often found to have retained its oxidised form, suggesting that it is not always bioavailable even when absorbed by the host [3, 8, 32]. The bacterial route increases bioavailability.

Further studies are required in order to determine whether folic acid supplementation affects the folate status of human gut microbes and whether this in turn impacts host health. Interestingly, there are several diseases associated with an increased abundance of folate-synthesizing gut bacteria, such as inflammatory bowel disease [33] and small intestinal bacterial overgrowth [34], but a causal relationship between bacterial folate and disease has not been established. The *abgT* gene is found in the genomes of several enteric pathogens, including *Enterobacter cloacae*, *Neisseria gonorrhoeae*, *Salmonella enterica*, *Shigella boydii* and *Staphylococcus aureus*, in addition to *E. coli.* Whilst there is much consideration about the consequences of folic acid supplementation [3, 9, 10], our work here indicates that folic acid supplement instability and bacterial metabolism are previously unexplored variables that may impact human health and thus warrant consideration in future reports and studies*.*

## Conclusions

The main route of uptake of the synthetic supplement folic acid by *C. elegans* is via *E. coli*, with a dependence on breakdown of folic acid. Folic acid supplements contain breakdown products, raising the possibility that this route could occur in humans. This route shortens the lifespan of *C. elegans* on folate-depleted *E. coli*, suggesting that there are circumstances in which this bacterial route could have negative consequences for human health.

## Methods

### Folates and related compounds

Folic acid, folinic acid, PABA-Glu, 5-formylTHF-Glu_3_, 5-methylTHF-Glu_3_ and methotrexate-Glu_6_ were obtained from Schircks, Switzerland. PABA, vitamin B12 and folic acid were purchased from Sigma Aldrich and folic acid supplement from Boots, UK.

### Culture conditions

DM was prepared as previously described [13], except that 10 nM vitamin B12 was added. Vitamin B12, folic acid and antibiotics were added post-autoclaving for agar plates. DM for liquid culture was filter sterilised. Following addition of 0.1 μM PABA to the liquid DM media, this was used to seed the plates in order to maintain bacterial growth (apart from growth experiments in Fig. 2). Then, 30 μL of 3 mL fresh overnight LB culture were used to inoculate 5 mL of DM (in 15 mL Falcon tubes). Kanamycin (25 μg/mL and 50 μg/mL of ampicillin if necessary) were added to both LB and DM pre-incubation. DM liquid cultures were incubated for 18 h at 37 °C and 220 RPM.

All strains were derived from the Keio collection [35] (Table 1). The Δ*abgT ΔpabA* double mutant was made using the P1 transduction protocol as described in [36]. The *abgT* over-expression plasmid (pJ128) [16] and empty vector (puc19) [37] were transformed into appropriate strains.

**Table 1:** *E. coli* strains used in this study

Strain	Genotype	Plasmid	Characteristics	Source
BW25113/pGreen 0029	WT	pGreen 0029	kan^r^	Virk et al. 2016 [13]
JW3323-1	*ΔpabA*	n/a	kan^r^	Baba et al. 2006 [25]
JW5822-1	*ΔabgT*	n/a	kan^r^	Baba et al. 2006 [25]
CM*abgTpabA*	*ΔabgT ΔpabA*	n/a	kan^r^	This study
CM1	WT	pUC19, pGreen 0029	kan^r^, amp^r^	This study
CM2	*ΔpabA*	pUC19	kan^r^, amp^r^	This study
CM3	*ΔabgT ΔpabA*	pUC19	kan^r^, amp^r^	This study
CM4	WT (*abgT* OE)	pJ128, pGreen 0029	kan^r^, amp^r^	This study
CM5	*ΔpabA* (*abgT* OE)	pJ128	kan^r^, amp^r^	This study
CM6	*ΔabgT ΔpabA* (*abgT* OE)	pJ128	kan^r^, amp^r^	This study

r= resistance

### *C. elegans* strains used

SS104 *glp-4(bn2)*, UF208 (WT) and UF209 *gcp-2.1(ok1004)* [13].

### *E. coli* preparation and growth assay

*E. coli* was prepared as follows for all *E. coli* and *C. elegans* experiments. A 30 μL aliquot of an overnight LB culture of *E. coli* was transferred into 5 mL of DM and incubated for 18 h at 37 °C and 220 RPM. Then, 100 μL of the DM culture was seeded onto DM agar plates and incubated at 25 °C for 4 days. *E. coli* was removed by pipetting 1 mL of M9 medium onto the plate and a glass spreader was used to scrape off the bacterial lawn. The bacterial suspension was pipetted into a 1.5 mL Eppendorf and the volume was recorded (v). Tubes were vortexed vigorously to obtain a homogenised solution. Finally, 100 μL were taken and diluted with 900 μL of M9 in a cuvette. A spectrophotometer was used to read bacterial growth at 600 nm. Bacterial growth was calculated by multiplying OD_600_ by the volume of the sample (v).

### *E. coli* folate extraction

Bacterial lawns were scraped from plates into microcentrifuge tubes using M9 solution and kept on ice. Volume (v) multiplied by the OD_600_ of the solution (diluted 1:5) gives a measure of the amount of material. Samples were concentrated in a cooled microcentrifuge and pellets were snap frozen in liquid nitrogen. Pellets were thawed and resuspended in a volume of ice-cold 90% methanol: 10% folate extraction buffer (FEB: 50 mM HEPES, 50 mM CHES, 0.5% *w*/*v* ascorbic acid, 0.2 M DTT, pH 7.85 with NaOH) in proportion to bacterial content (37.5 × OD_600_ × v). FEB was spiked with 10 nM methotrexate-Glu_6_ as an internal standard. Samples were vortexed vigorously and left on ice for 15 min before centrifugation in a cooled microcentrifuge for 15 min at full speed. Supernatants were used for analysis.

### Folate LC-MS/MS analysis

Folates were detected by multiple reaction monitoring (MRM) analysis using a SCIEX QTRAP 6500 instrument. MRM conditions for folic acid, PABA, PABA-Glu, 5-Me-H_4_PteGlu_3_ (5-methylTHF-Glu_3_) and 5/10-CHO-H_4_PteGlu_3_ (formyl THF_3_) were optimised by infusion of standards into the instrument. The optimised conditions for –Glu_3_ folates were applied to other higher folates using MRM transitions as described by Lu et al. [38]. Further confirmation of identity for folates of interest was achieved by performing enhanced product ion scans and comparing the fragment spectra with known standards.

The QTRAP 6500 was operated in ESI+ mode and was interfaced with a Shimadzu Nexera UHPLC system. Samples were separated using a Thermo PA2 C18 column (2.2 μm, 2.1 × 100 mm) with a gradient of 0.1% formic acid in water (mobile phase A) and acetonitrile (mobile phase B). Samples were maintained at 4 °C and 2 μL aliquots were injected. The column was maintained at 40 °C with a flow rate of 200 μL/min, starting at 2% B, held for 2 min, with a linear gradient to 100% B at 7 min, held for 1 min, before a 7 min re-equilibration step at 2% B necessary for consistent retention times. The column eluate flow to the MS was controlled via the QTRAP switching valve, allowing analysis between 4 and 8 min to minimise instrument contamination. Folates were quantified with reference to external standards and matrix effects were assessed by spiking of standards into extracted samples.

### Lifespan analysis

Survival analyses were performed as described [12]. *glp-4(bn2)* worms were maintained at 15 °C and shifted to 25 °C at the L3 stage. At the L4/young adult stage, animals were placed on bacteria under the experimental conditions. All lifespan data is provided in Additional file 1: Table S1. Statistical significance was determined using Log Rank and Wilcoxon tests of the Kaplan–Meier survival model.

## Additional files

Additional file 1: **Table S1.** Full analysis of lifespan conditions including statistical analysis. (XLSX 46 kb)

Additional file 2: **Figure S1.** The *E. coli ΔabgT* deletion has no effect on *C. elegans* lifespan survival curves of *C. elegans glp-4(bn2)* on WT *E. coli* and *ΔabgT* mutant*.* See Additional file 1: Table S1 for further details. (PDF 72 kb)

Additional file 3: **Table S2.** Data corresponding to Fig. 4 in the main text. (XLSX 50 kb)

Additional file 4: **Table S3.** Data corresponding to Fig. 5 in the main text. (XLSX 10 kb)
