# Supplementary figures and images for: A bacterial route for folic acid supplementation
Source: BMC Biol. 2018 Jun 15;16:67. doi: 10.1186/s12915-018-0534-3 (PMC6002978; doi:10.1186/s12915-018-0534-3)

Supplementary Figure 1

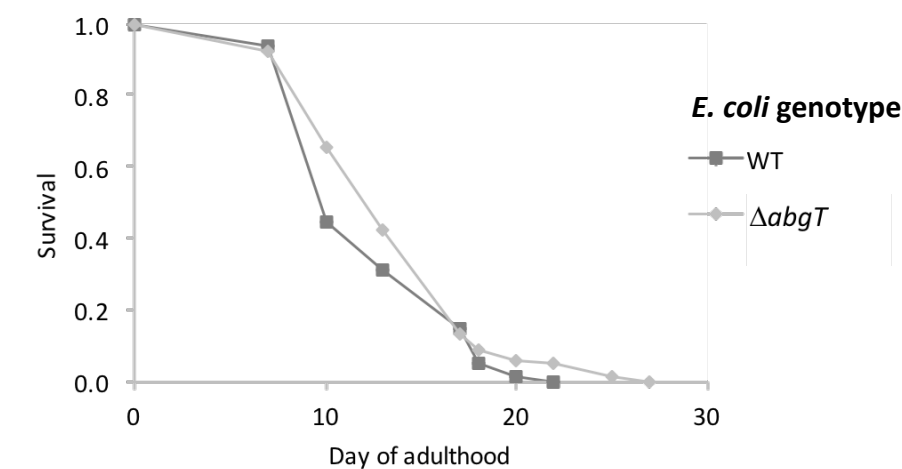

Supplement: Supplementary file 2 — Figure S1. The E. coli ΔabgT deletion has no effect on C. elegans lifespan survival curves of C. elegans glp-4(bn2) on WT E. coli and ΔabgT mutant. See Additional file 1: Table S1 for further details. (PDF 72 kb) [file 12915_2018_534_MOESM2_ESM.pdf]
